# Efficacy and safety of butylphthalide in secondary prevention of stroke: study protocol for a multicenter, real world trial based on Internet

**DOI:** 10.1186/s12883-022-02815-x

**Published:** 2022-08-19

**Authors:** Junchao Lv, Di Zhao, Gang Zhao, Zhen Xie

**Affiliations:** 1 Xijing Hospital, Fourth Military Medical University, No. 127 Changle west road, Xincheng district, Shaanxi Province 710000 Xi’an, China; 2 School of Medicine, Northwestern University of China, No. 229, Taibai North Road, Beilin District, Shaanxi Province 710000 Xi’an, China

**Keywords:** Ischemic stroke, Secondary prevention, Butylphthalide, Multicenter, Real-world

## Abstract

**Background:**

As one of the leading causes of morbidity and mortality, stroke and its recurrence has attracted more and more attention. Dl-3-n-butylphthalidle(NBP) has been widely used for treating acute ischemic stroke in China and shows a great clinical effect. NBP plays a role in different pathophysiological processes in the treatment of ischemic stroke, including antioxidants, anti-inflammatory, anti-apoptotic, anti-thrombosis, and mitochondrial protection. Many randomized, double-blind, placebo-controlled, multicenter clinical trials suggest that NBP is a safe and effective treatment for ischemic stroke. To sum up, the current research is mainly focused on the short-term treatment of stroke patients with RCT (randomized controlled trial). Therefore, we designed this study to confirm the role of butylphthalide in secondary stroke prevention in the real world.

**Methods:**

This study will be a multicenter, prospective real-world trial. We would recruit 8000 patients with ischemic stroke from 78 public hospitals in China. All participants will be allocated to one of two parallel treatment groups according to their own wills: (1) butylphthalide group: 0.2 g of butylphthalide capsules three times daily plus routine treatment (aspirin 50-300 mg/d, clopidogrel 75 mg/d, etc.); (2) control group: routine treatment (aspirin 50-300 mg/d, clopidogrel 75 mg/d, etc.). Treatment duration is 90 consecutive days or more. The primary outcome is recurrence rate of stroke within 1 month, 3 months, 6 months and 1 year in butylphthalide group and control group. The secondary outcomes included NIHSS score, the mRS score, other clinical cardiovascular events within one year (sudden death / myocardial infarction / arrhythmia / heart failure, etc.), and adverse events of patients in groups. NIHSS will be captured in the first month after discharge, and the others will be captured at the same time points as the primary end point.

**Discussion:**

This trial will be exploring the efficacy and safety of butylphthalide in secondary prevention of ischemic stroke to expand the scope of application of butylphthalide soft capsules and provide new ideas for enriching the secondary prevention of stroke.

**Trial registration:**

Chinese Clinical Trial Registry (ChiCTR). Trial registration number: ChiCTR2000034481. Registered on 6 July 2020, http://www.chictr.org.cn/showproj.aspx?proj=55800

**Supplementary Information:**

The online version contains supplementary material available at 10.1186/s12883-022-02815-x.

## Administrative information

**Table:** 

Title {1}	Efficacy and safety of butylphthalide in secondary prevention of stroke: study protocol for a multicenter, real world trial based on Internet
Trial registration {2a and 2b}	Chinese Clinical Trial Registry (ChiCTR). Trial registration number: ChiCTR2000034481. Registered on 6 July 2020, http://www.chictr.org.cn/showproj.aspx?proj=55800 The Universal Trial Number (UTN) is U1111-1256–0166
Protocol version {3}	20,200,821
Funding {4}	Shijiazhuang Pharmaceutical Group Pharmaceutical Co. Ltd Shaanxi Provincial Natural Science Basic Research Program (NO.2019JQ-251) Xi’an International Medical Center Hospital Hospital Level Project (NO.2020ZD007)
Author details {5a}	Junchao Lv Xijing Hospital, Fourth Military Medical University Xi’an 710,000, Shaanxi Province, China https://996524996@qq.com Di Zhao Xijing Hospital, Fourth Military Medical University Xi’an 710,000, Shaanxi Province, China https://zhaoditieren@163.com Correspondence author: Zhen Xie* School of Medicine, Northwestern University of China, No. 229, Taibai north road, Beilin district, Xi’an710000, Shaanxi province, China https://xiezhenhh@163.com Gang Zhao 1. Xijing Hospital, Fourth Military Medical University, No. 127 Changle west road, Xincheng district Xi’an 710000, Shaanxi Province, China School of Medicine, Northwestern University of China, No. 229, Taibai north road, Beilin district, Xi’an 710000, Shaanxi province, China https://zhaogang@nwu.edu.cn
Name and contact information for the trial sponsor {5b}	This study is funded by Shijiazhuang Pharmaceutical Group Pharmaceutical Co. Ltd.(Shijiazhuang, China), Shaanxi Provincial Natural Science Basic Research Program (NO.2019JQ-251), and Xi’an International Medical Center Hospital Hospital Level Project (NO.2020ZD007)
Role of sponsor {5c}	The funding bodies have no role in the trial design or interpretation of the data

## Introduction {6}

### Background {6a}

Stroke is a common cerebrovascular disease, which is characterized by high incidence, high disability rate and mortality. Acute ischemic stroke (AIS) accounts for 60% to 80% of stroke. It is a group of clinical syndromes caused by brain tissue blood supply disorder, resulting in ischemic and hypoxic necrosis, and then neurological dysfunction [1]. According to data from the China National Stroke Registry, the recurrence rate of acute stroke patients in China is 17.7% in the first year. The 5-year cumulative recurrence rate exceeds 30% [2]. The American Heart Association Council predicts that another 3.4 million American adults ≥ 18 years of age will have a stroke in 2030. The prevalence rate will increase by 20.5% compared with 2012 [3]. In addition, stroke places a great deal of economic burden on patients, their families as well as the entire society. From 2015 to 2035, the total number of direct medical strokes and related costs are expected to more than double, from $36.7 billion to $94.3 billion [3]. Therefore, how to reduce the recurrence of stroke and improve the prognosis is a hot topic for medical workers all over the world.

NBP has been widely used for treating acute ischemic stroke in China and shows a great clinical effect [4]. According to the Chinese Guidelines for Evaluation and Intervention of Collateral circulation in Ischemic Stroke 2017, interventions for collateral circulation include non-drug interventions, such as external counterpulsation (IIb recommendation, Grade C evidence); and drug interventions, such as NBP (IIa recommendation, Level B evidence) [5]. According to a meta-analysis in 2019, researchers found evidence that the combination of NBP and conventional neuroprotective agents can improve functional recovery 90 days after ischemic stroke compared with traditional drugs alone [6]. NBP is a safe and effective treatment for progressive cerebral infarction (PCI). A randomized, double-blind, controlled study was conducted on the effectiveness and safety of NBP in the treatment of PCI (Zhang et al., 2017). The primary endpoints were the National Institutes of Health Stroke Scale (NIHSS) score before treatment and at 7, 14, 21, and 30 days after treatment, and Barthel Index (BI) evaluation on the 90th day of treatment. The result shows that NBP can improve the prognosis and reduce the burden of daily life of PCI patients [7]. Other randomized, double-blind, placebo-controlled, multicenter clinical trials suggest that NBP may be a safe and effective treatment for ischemic stroke [8–10]. In addition, a Phase II clinical trial of NBP soft capsules for patients with ischemic stroke began in the United States in 2017 (NCT02905565) [5]. To sum up, the current research is mainly focused on the short-term treatment of stroke patients with RCT. Therefore, we designed this study to confirm the role of butylphthalide in secondary stroke prevention in the real world.

### Rationale

NBP was originally extracted from the seeds of Apium Graveolens Linn. It is an effective ingredient of Chinese herbal medicine that used to treat epilepsy [11]. However, it has been found that the effective dose and the toxic dose in treating epilepsy are close, which has a greater safety risk. Therefore, the focus of research has shifted from epilepsy to stroke. Subsequent studies have shown that in the rat ischemic stroke model, NBP can reduce infarct volume, neuronal cell death and brain edema, and preserve the blood–brain barrier [11–17]. A study compared the incidence of stroke, the volume of the brain lesion, patency of the microvessels by FITC-dextran perfusion and the number of microvessels by immunohisochemical detection of vwF between NBP pretreatment group and control group. The incidence of ischemic stroke and the volume of the infarct were decreased, and the perfused microvessels were increased with NBP pretreatment [18]. In addition, NBP reduced glial fibrillary acidic protein (GFAP)-positive astrocytes induced by chronic cerebral ischemia, so as to play a protective effect on hippocampal injury [19]. NBP plays a role in different pathophysiological processes in the treatment of ischemic stroke, including antioxidants, anti-inflammatory, anti-apoptotic, anti-thrombosis, and mitochondrial protection [4, 20]. A study showed that NBP could display strong activity in inhibiting the arachidonic acid (AA)- and adenosine diphosphate (ADP)-induced platelet aggregation in vitro. Furthermore, NBP could release moderate levels of NO and H2S, which would be beneficial in improving cardiovascular and cerebral circulation [21]. Butylphthalide also increases vasodilation and inhibits platelet aggregation [22]. NBP has been confirmed to increases VEGF expression and decreases poststroke inflammation and MMP-9 expression in animal models of stroke [23, 24]. In view of its powerful therapeutic effect on ischemic stroke in animal models, the State Food and Drug Administration of China approved it for clinical trials. The Phase IV clinical research trial was completed in February 2005. NIHSS, Barthel index (BI) and Activities of Daily living scale (ADL) were used as the end points of the study. Total effective rate = (Basic recovery cases + significant improvement cases)^a^ / total number of cases × 100%. And the total effective rate of butylphthalide was 78.2% [25, 26]. A research investigated the effects of dl-3-n-butylphthalide (NBP) on the level of circulating endothelial progenitor cells (EPCs) and clinical outcome in patients with acute ischemic stroke (AIS).The results showed that the levels of circulating EPCs on days 14 and 30 were significantly higher in the NBP group than in the control group. In contrast, NIHSS score was notably lower in NBP group on day 14, 30 and day 90. The mRS score in the NBP group was lower on day 90 [27].

*a:Basic recovery: the score of neurological impairment decreased by 91%-100%, and the degree of disability was 0;*

*Significant progress: the score of neurological impairment decreased by 46% to 90%, and the degree of disability was 1;*

*Progress: the score of neurological impairment decreased by 18% to 45%;*

*No change: the score of neurological impairment decreased or increased by less than 17%;*

*Deterioration: the score of neurological impairment increased by more than 18%.*

### Explanation for the choice of comparators {6b}

Doctors will make medication plans for secondary prevention according to their parents’ condition when they are discharged, and recommend (rather than forcing) them to take butylphthalide. We will manage patients through Internet means such as WeChat Mini Programs, and record their medication regularly. At the end of the whole study cycle, we will take patients who take NBP for 3 months as NBP group, and patients who refused to take butylphthalide or poor drug compliance^b^ as the control group. In addition, we will faithfully record other drugs except butylphthalide soft capsules.

*b:According to the formula: “medication compliance* = *actual number of tablets / number of tablets to be taken* × *100%”, we calculate the patient’s drug compliance. 80% to 120% will be considered as good compliance, while* < *80% or* > *120% will be considered poor compliance.*

## Objectives {7}

The objective of this trial is to confirm the efficacy and safety of butylphthalide on secondary prevention of stroke patients through follow-up of the experimental group and the control group. It provides clinical evidence for NBP to prevent and treat stroke, and has certain significance for improving treatment and prognosis of patients.

## Trial design {8}

**Fig. 1:**
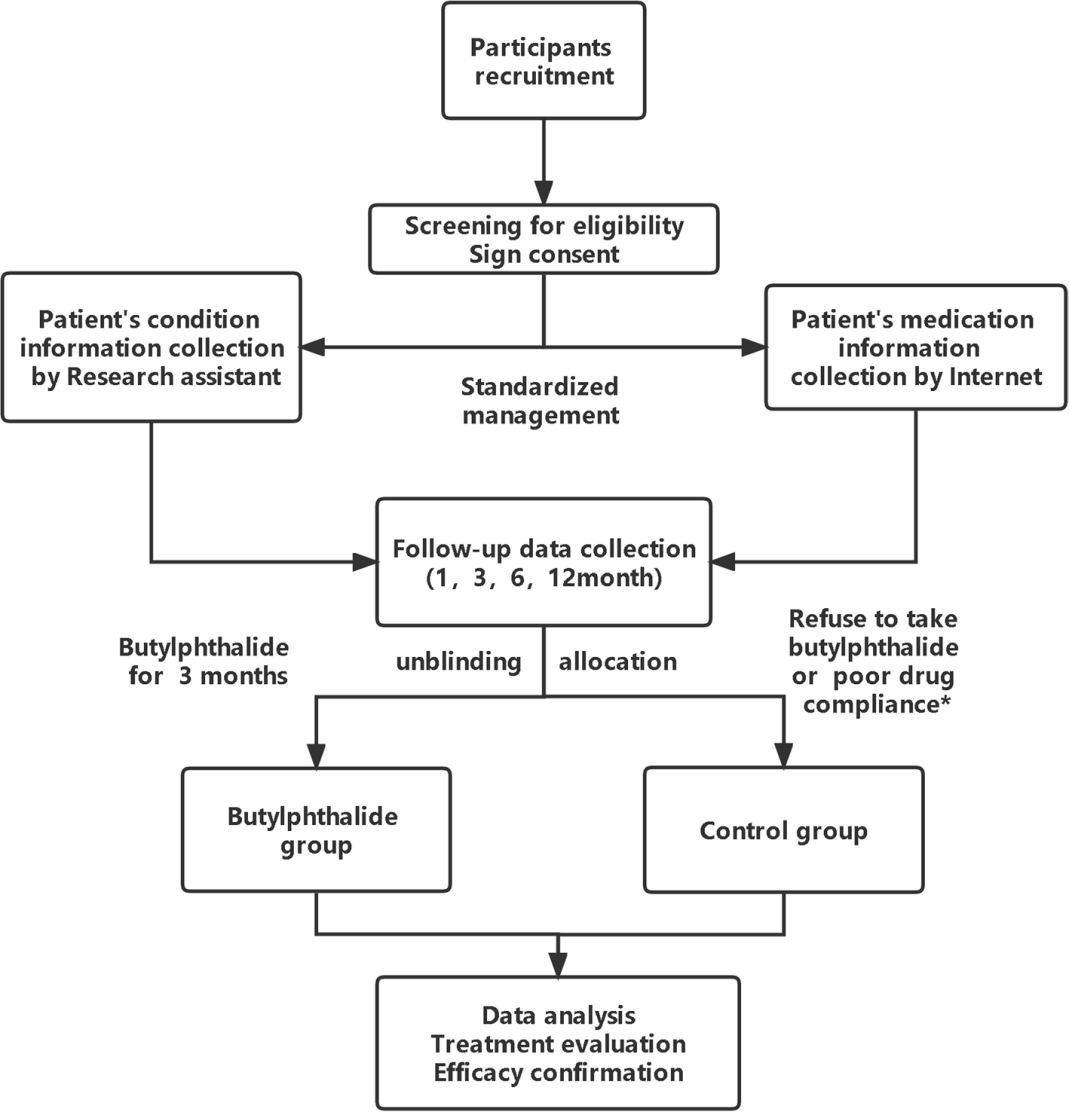
The flow chart of the trial. *: According to formula: “medication complianece = actual number of tablets / nunmber of tablets to be taken × 100%”, we calculate the patient's drug compliance. 80% to 120% will be considered as good compliance, while < 80% or > 120% will be considered poor compliance

This study is an open-label, noninferiority, two-armed, prospective, multicenter, real world trial conducted at Northwest University in Xi’an, China. The participants will be recruited from 78 hospitals across mainland China. Eligible participants will be assigned to the butylphthalide group or the control group according to their own wills. Every researcher at each study center is scheduled to recruit patients from December 2020 to June 2023. The flow chart of the trial is shown in Fig. 1.

## Methods: participants, interventions and outcomes

### Study setting {9}

We will recruit 8000 subjects from the following 78 hospitals. The list of recruitment hospitals can be found in supplementary material 1. All subjects in each treatment arm of the study will participate voluntarily after informed consent is obtained. Follow-up will be arranged at months 1, 3, 6, 12 after discharge. The project team leader communicates with the researchers of each center on a regular basis and conducts study visits in all research centers.

### Eligibility criteria {10}

#### Subject recruitment/screening

Cases will be recruited from the neurology department of 78 hospitals in China, which have a high level of diagnosis and treatment and are capable of performing CT, MRI, and other examinations. The doctor in charge of each trial center is responsible for the enrollment and follow-up of patients. Before enrollment, patients can be included only with informed consent. The follow-up is expected to be one year, and each follow-up is used to confirm whether the patient meets the exclusion criteria or not.

To ensure the accuracy of the diagnosis and treatment of stroke, the recruitment criteria for clinicians are as follows:

1. Neurology residents in 78 hospitals;
2. Complete at least 5 years of clinical medicine professional study and 3 years of standardized training for resident physicians;
3. Complete the one-hour training course for this study. One-hour training includes:
  - The basic introduction of this research protocol;
  - The basic introduction of this research protocol;
  - Instructions for the use of WeChat Mini Programs;
  - Other considerations;
  - Question and answer.

### Diagnostic criteria

The diagnosis of ischemic stroke will have to meet the following criteria [28]:

1. Acute onset. The symptoms peak in more than 10 h or 1–2 days [29];
2. Neurologic dysfunction of any severity consistent with focal brain ischemia(such as weakness or numbness on one side of the face or body, language disorder, etc.), and few may have comprehensive neurologic dysfunction;
3. Imaging/laboratory confirmation of an acute vascular ischemic pathology;
4. Exclude non-vascular causes;
5. Brain CT/MRI excluded cerebral hemorrhage.

### Inclusion criteria

Subjects will be included in our study if they satisfy the following criteria:

1. Aged between 18 and 80 years;
2. Hospitalized patients with ischemic stroke diagnosed by clinical symptoms or signs. The clinical manifestations are acute onset of local or diffuse brain injury. And neurological impairment conformed to the characteristics of vascular distribution;
3. Imaging/laboratory confirmation of an acute vascular ischemic pathology;
4. Voluntarily provide written and informed consent.

### Exclusion criteria

Subjects will be excluded in our study if they satisfy the following criteria:

1. Discharged, transferred to another department or died within 24 h;
2. Severe heart, liver, kidney and other organs dysfunction or severe systemic diseases expected to survive less than one year;
3. Can not be followed up continuously;
4. Allergic to butylphthalide;
5. Pregnancy, lactation or planned pregnancy;
6. Severe mental disorders or dementia.

All patients will be recruited voluntarily and informed consent should be signed. If it is not in the patient’s interest, the patient could withdraw from the study. The reason for each withdrawal patient should be assessed and recorded in the electronic case report form (eCRF).

### Who will take informed consent? {26a}

Patients diagnosed with acute ischemic stroke or their authorized agents will read the informed consent form carefully before joining the study. The researchers will interview them and help them fully understand the research content and potential risks. The informed consent form can be found in supplementary material 2.

### Additional consent provisions for collection and use of participant data and biological specimens {26b}

Not applicable.

## Interventions

### Intervention description {11a}

1. Butylphthalide group: 0.2 g of butylphthalide capsules three times daily plus routine treatment (aspirin 50-300 mg/d, clopidogrel 75 mg/d, etc.).
2. Control group: routine treatment (aspirin 50-300 mg/d, clopidogrel 75 mg/d, etc.).

After discharge, all the subjects keep in touch with the doctors in charge by Wechat group or Mini Program and take medicine regularly to achieve high medication compliance. We plan to communicate with patients at a fixed time every week (for example, 8 p.m. Thursday). At that time, patients could communicate and ask any questions about the study in the WeChat group. Treatment duration is 90 consecutive days or more.

### Criteria for discontinuing or modifying allocated interventions {11b}

If patients have any discomfort, new changes in their condition, or any unexpected circumstances during the study, whether related to the study or not, they should inform their doctors in time, and doctors will make a judgment and give appropriate medical treatment.

### Strategies to improve adherence to interventions {11c}

This study is based on the network platform for follow-up by a variety of means to improve patient adherence. The doctor in charge of the patients will prescribe prescription and instruct them how to use the drug by Wechat Mini Programs. The research assistant of each research center will invite the patients to the Wechat group, remind them of the medication time, make an appointment for follow-up, and solve any questions raised by the patients in the Wechat group in time. Medication compliance will be calculated as the actual taken amount of study medication documented in a participant’s daily diary divided by the total amount planned for the participant.

### Relevant concomitant care permitted or prohibited during the trial {11d}

Medications applied to treat the underlying diseases (such as diabetes, hyperlipidemia and hypertension and others) are permitted. However, any medications used in this study should be documented in detail in case report form (CRF), including name, dose, dosage regimen, indication, and treatment period.

### Provisions for post-trial care {30}

If an adverse event occurs in a clinical trial, the committee of medical experts will determine whether it is related to therapeutic drugs. The organizer will provide treatment fees and corresponding economic compensation for the test-related damage in accordance with the provisions of “Drug Clinical trial quality Management Code of China”.

### Outcomes {12}

#### Primary outcome

##### Recurrence rate of stroke

The proportion of recurrent stroke in patients with ischemic stroke.

Recurrent stroke is defined as a new neurological deficit at least 24 h after the incident stroke according to the World Health Organization definition excluding edema, mass effect, brain shift syndrome or hemorrhagic transformation, and procedure-related strokes [30].

Our researchers will follow up patients and compare the recurrence rate of stroke between the butylphthalide group and the control group at month1, 3, 6 and 12. We will ask the patient’s condition in detail in every follow-up after discharge. If the stroke relapses, the researchers will record the time of recurrence, location of the lesion, TOAST classification, occlusive vessels, sequelae, complications, other diagnoses and related examination results. The researchers will discuss with his doctor in charge to carefully identify whether the patient has a relapse or initial progression.

#### Secondary outcome

##### NIHSS score

NIHSS score is a practical tool in the clinic to evaluate the degree of neurological impairment. NIHSS score of 42 points and 11 items, which takes into account the symptoms of anterior circulation and posterior circulation, is an objective semi-quantitative evaluation tool of stroke severity. It has been widely used in many international multicenter randomized controlled studies, and is recognized as a grading system for stroke patients’ severity with good repeatability. Generally, NIHSS score needs to be scored face to face between doctors and patients. However, due to various reasons, patients usually cannot return to the hospital for follow-up, resulting in a large number of dropouts. Therefore, in this study, Wechat is used to follow up the discharged patients. The researchers make an appointment with the patient for follow-up in advance and score the patient by video calls.

##### MRS score

The modified Rankin scale is used to measure the recovery of neurological function in patients with stroke. It has the following advantages:

1. The operation is simple, and the score can be obtained through simple inquiry;
2. As the effective evaluation index of functional disability level, mRS has good reliability and authenticity, and the standard mRS evaluation method is direct interview between the evaluator and the patient.
3. MRS has no physical examination related content, so remote assessment can be realized by phone or questionnaire.

##### Adverse events

Adverse events include emerging clinical cardiovascular events within one year (sudden death/ myocardial infarction/ arrhythmia/ heart failure, etc.), and other adverse reactions of patients in the trial group. During the follow-up period, we will add tests for other adverse reactions such as liver and kidney function, bleeding and blood coagulation.

### Participant timeline {13}

Schedule of the study procedures is shown in Table 1.

**Table 1:**
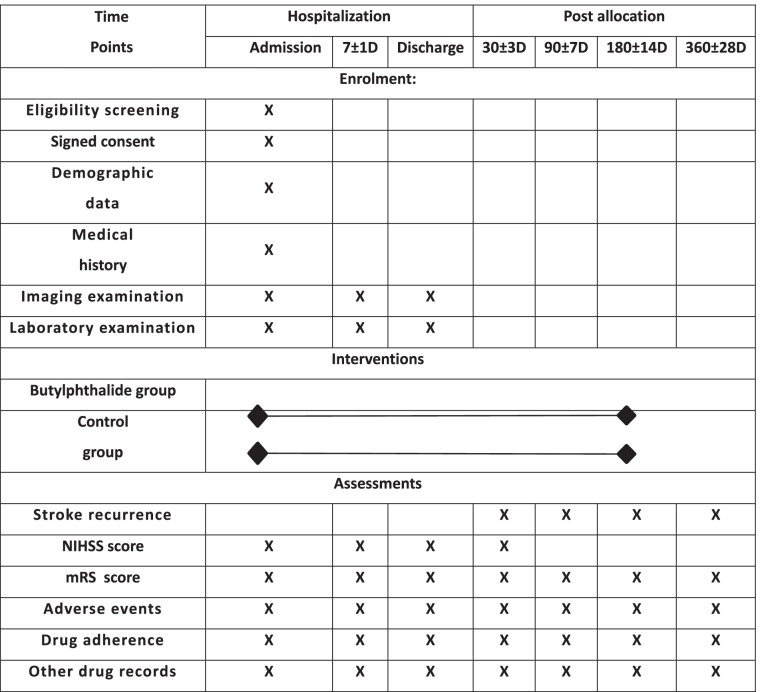
Schedule of study procedures

### Sample size {14}

The sample size calculation was conducted using the PASS software version 25 (NCSS, LLC. Kaysville, Utah, USA). Previous studies have shown that one-year recurrence rate of stroke patients in China was 17.7% [1]. The underlying assumption was a decline of the recurrence rate of stroke from 17.7% to 14.7%. The sample size of the experimental group and the control group was about 3168 with at least 90% power and an overall two-sided alpha level of 0.05. Taking approximate dropouts of 15% into account, the number of participants recruited was estimated to be 3727 each group. Therefore, 8000 patients will be recruited for this trial.

### Recruitment {15}

We will recruit 8000 subjects from the following 78 hospitals.The list of recruitment hospitals can be found in supplementary material 1.

## Assignment of interventions: allocation

### Sequence generation {16a}

This study is a real-world trial, with subjects assigned to this trial based on their actual conditions and wishes.

### Concealment mechanism {16b}

This study is an open-label, real world trial. So the patients and doctors know the exact grouping. We will further upgrade WeChat Mini Programs to automatically remind patients of medication and send forms for patients to fill in their medication. The research assistant is mainly responsible for collecting information about the patient’s condition, and do not know their medication. At the end of the whole research cycle, biostatisticians will unblind and analyze the data.

### Implementation {16c}

Doctors will recruit patients with ischemic stroke who meet the inclusion criteria, and give them standardized treatment. Research assistants invite the patients into the patient management Wechat group and collect the baseline information of the patients. Biostatisticians are responsible for collecting and analyzing the uploaded data of each center. It embodies the characteristics of real world research in the process of implementation.

### Real world research settings

#### Intervention measures

In order to better reflect the efficacy of butylphthalide soft capsules in the real world, doctors will make medication plans for secondary prevention according to their condition when they are discharged, and recommend (rather than forcing) patients to take butylphthalide. The researchers will not take other interventions for the secondary prevention of patients.

#### Data collection

The research assistants will follow up the patient at 1, 3, 6, 12 months after discharge through the Internet(such as WeChat Mini Programs), and faithfully collect the patient’s condition. In addition, WeChat Mini Programs will send CRF forms to patients to obtain their medication information.

#### Unblinding and allocation

At the end of the study cycle, the researchers will reveal the actual medication information of the patients, taking patients with good butylphthalide compliance as the butylphthalide group, and patients without taking butylphthalide and patients with poor compliance as the control group, and compare the difference of outcomes between the two groups.

## Assignment of interventions: blinding

### Who will be blinded {17a}

A research assistant will be responsible for following up the participants in every research center. Researchers, research assistants and statisticians will be masked to allocate treatment throughout the treatment period. No blindness is allowed only in the event of serious adverse events (SAE) or emergency rescue. Statistical analysis will be conducted by independent statisticians who only know the group code and do not know the corresponding interventions.

### Procedure for unblinding if needed {17b}

No blindness is allowed only in the event of serious adverse events (SAE) or emergency rescue. When serious adverse reactions occur, the doctor in charge of the center who knows the the exact grouping of patients will deal with them immediately and inform research assistant to report them in time.

## Data collection and management

### Plans for assessment and collection of outcomes {18a}

The patient’s baseline information will be collected by the research assistant while in hospital. During follow-up period, the doctor regularly makes video calls with the patient, conducts detailed consultations with the patient, collects the patient’s follow-up information and fills it in the CRF form. Afterwards, all research data will be sorted by the research assistants of each center and uploaded to the EDC platform. The data management experts of the research center conduct a unified review of the uploaded data to ensure the accuracy and completeness of the data. We will remove those centers with poor data quality, missing follow-up information, and high dropout rates from continuing to participate in the study.

### Plans to promote participant retention and complete follow-up {18b}

In order to encourage the subjects to complete follow-up and improve their compliance, all subjects in each center will be assigned to the patient management Wechat group by the the doctor in charge. In Wechat group, doctors and research assistants can record the enrollment time of patients, make an appointment for follow-up, and answer questions in time. In addition, we have developed a WeChat follow-up mini program in which patients can sign in daily and write their own stroke diary.

### Data management {19}

All data will be documented on CRF that records all information at baseline and follow-up, and immediately uploaded in the database through the EDC (Electronic Data Capture) system. The CRF includes demographic data, living habits, medical history, follow-up data, and adverse events. The data will be entered into the database by a designated operator and checked repeatedly by the research center. The whole process is anonymous. In order to protect the privacy of patients, their registration number will be used instead of their real name when filling out the form. Dropouts and adverse events (adverse events) will be recorded in a timely manner. In order to ensure the high quality of the trial, members of the Quality Management Committee will train all researchers involved in the trial in accordance with standard operating procedures before the trial starts.

### Confidentiality {27}

The whole process is anonymous. In order to protect the privacy of patients, their registration number will be used instead of their real name when filling out the form. The patient’s personal information (such as home address, telephone number, Wechat account and others)will be kept strictly confidential throughout the study. All hard copy CRF will be kept by authorized researchers only. Data such as eCRF is stored in the dedicated Aliyun server of Northwest University. We will minimize the number of data processing personnel to ensure data confidentiality.

### Plans for collection, laboratory evaluation and storage of biological specimens for genetic or molecular analysis in this trial/future use {33}

Not applicable.

## Statistical methods

### Statistical methods for primary and secondary outcomes {20a}

The Kaplan–Meier method is used to draw the survival analysis curve of the percentage of patients with recurrent stroke within 1 month, 3 months, 6 months and 1 year of follow-up. The LogRank method is used to compare the statistical difference in the survival curve between the two groups. The COX proportional regression model is used to calculate the risk ratio (hazards ratios) and 95% CI.

The measurement data (such as NIHSS score and modified Rankin score) is expressed by x ± s, and t-test or Wilcoxon rank sum test is used for comparison between groups. The counting data (such as clinical efficacy and adverse reactions of the two groups) is expressed by chi-square test or Fisher exact test. The subgroup analysis is carried out according to sex, age, medical history and type of stroke.Propensity score matching (PSM) is taken to ensure that the covariates are balanced and comparable. A two-sided *P* value of 0.05 or less is considered statistically significant. All the analyses are done with SPSS software 18.0 or based on R software 3.4.3.

### Interim analyses {21b}

Not applicable.

### Methods for additional analyses (e.g. subgroup analyses) {20b}

Not applicable.

### Methods in analysis to handle protocol non-adherence and any statistical methods to handle missing data {20c}

Not applicable.

### Plans to give access to the full protocol, participant level-data and statistical code {31c}

Not applicable.

## Oversight and monitoring

### Composition of the coordinating centre and trial steering committee {5d}

The School of Medicine of Northwestern University of China is the coordination center of this study, which is responsible for the supervision and ethical review of the whole research process to ensure the proper implementation of this trial. When the trial starts, the coordination center will coordinate and monitor 78 sub-centers. The coordinating center will track trial progress at least once a month by networking, phone call, email or onsite monitoring. Every research assistent is responsible for coordinating and organizing the enrolment, informed consent, and follow-up procedures at their local sites. There are no stakeholders and public participation groups in this trial.

### Composition of the data monitoring committee, its role and reporting structure {21a}

The study will be monitored by an independent data monitoring committee (DMC). The monitor regularly conducts onsite monitoring and reviews the CRFs to ensure participants’ rights and interests, compliance with the trial protocol, source data validity, data accuracy, and data integrity.

### Adverse event reporting and harms {22}

If an adverse event occurs in a clinical trial, the committee of medical experts will determine whether it is related to basic therapeutic drugs. The organizer will provide treatment fees and corresponding economic compensation for the test-related damage in accordance with the provisions of “Drug Clinical trial quality Management Code of China”.

### Frequency and plans for auditing trial conduct {23}

An independent auditor will audit this trial, regarding trial conduct and compliance with the protocol, standard operating procedures, good clinical practice, and the applicable regulatory requirements.

### Plans for communicating important protocol amendments to relevant parties (e.g. trial participants, ethical committees) {25}

Any major amendments need to be reported to the Medical Ethics Committee of Northwestern University of China before they can be implemented. And DMC will be responsible for data monitoring, interim analysis, assessment of adverse events and review of core trial procedures and documentation, discussion of any revisions to the main study protocol, and making the final decision to terminate the trial.

## Dissemination plans {31a}

The full protocol and datasets analyses of the current study are available from the corresponding author on reasonable request. The primary investigators and sponsor will communicate the trial results to participants and all the investigators involved. The trial results will be submitted to national or international conferences or peer-reviewed journals with the sponsor’s permission.

## Discussion

A systematic review found evidence suggesting that the combination of NBP and conventional neuroprotective agents improves functional recovery at 90 days after ischemic stroke compared with conventional drugs alone [6]. And many randomized, double-blind, placebo-controlled, multicenter clinical trials also suggest that NBP may be a safe and effective treatment for ischemic stroke [7–10]. This trial will be exploring the efficacy and safety of butylphthalide in secondary prevention of ischemic stroke, in order to expand the application of butylphthalide soft capsules and provide new evidence for enriching the secondary prevention of stroke.

In addition, this trial is a real-world trial highlighting the practicability of the research results. We have a larger sample size and a longer follow-up time, which can better reduce the sampling error and more easily detect other adverse events. This research can better represent the target population, and its treatment methods are close to the actual treatment [31], which can better reflect the effectiveness of butylphthalide in the real world.

The most important feature of this study is to use the network platform to complete the follow-up. In previous studies, it is difficult for patients to complete NIHSS score or mRS score in follow-up by returning to the hospital, resulting in a large number of dropouts and incomplete information. In this study, researchers score the mRS and NIHSS of the subjects by making a video call with them. So it could eliminate the inconvenience of patients returning to hospital for follow-up. In addition, by Wechat group, Wechat Mini Programs and other ways, we could manage patients, remind patients of medication time, guide patients how to use drugs, and greatly increase patients’ compliance with medication. It is critical for the management of medical big data in the future in China. In the future, we may develop artificial intelligence, machine learning and other means to standardize scoring.

## Trial status

This trial is in the recruitment phase. Patient recruitment begins in December 2020 and is expected to be completed in June 2023.

## Supplementary Information

**Additional file 1:** **Appendix 1.** The list of recruitment hospitals.

**Additional file 2:** **Appendix 2.**The informed consent form.

## Data Availability

The datasets generated and analyzed during the current study are available from the corresponding author upon request.

## Abbreviations

NBP: Dl-3-n-butylphthalidle
RCT: Randomized controlled trial
NIHSS: National institutes of health stroke scale
MRS: Modified Rankin Scale
ChiCTR: Chinese Clinical Trial Registry.
SPIRIT: Standard Protocol Items
SAE: Serious adverse events
CRF: Case report form
eCRF: Electronic case report form
EDC: Electronic Data Capture System
AEs: Adverse events
DMC: Data monitoring committee
