# Supplementary material for: Efficacy and safety of butylphthalide in secondary prevention of stroke: study protocol for a multicenter, real world trial based on Internet
Source: BMC Neurol. 2022 Aug 19;22:305. doi: 10.1186/s12883-022-02815-x (PMC9389750; doi:10.1186/s12883-022-02815-x)
Supplement: Supplementary file 1 — Additional file 1: Appendix 1. The list of recruitment hospitals. [file 12883_2022_2815_MOESM1_ESM.docx]

**Appendix 1. The list of recruitment hospitals**

Xi'an International Medical Center hospital, Xi'an No.1 hospital, Xi'an No.3 hospital, Xi'an Gaoxin hospital, Shenmu hospital, Xi'an Shaanxi traditional Chinese Medicine hospital, Xi'an traditional Chinese Medicine hospital Xi'an Friendship hospital, Shangluo Danfeng people's hospital, Shangluo Luonan people's hospital, Shangluo Shanyang people's hospital, Shangluo Shanyang traditional Chinese Medicine hospital, Shangluo Shangnan traditional Chinese Medicine hospital, Shangluo Shangzhou District people's hospital, Shangluo City Zhen'an people's hospital, Shangluo City Zhenan traditional Chinese Medicine hospital, Tongchuan Yaozhou District people's hospital, Tongchuan Yijun County people's hospital, Weinan City Baishui County hospital, Weinan City Dali County hospital, Weinan City Dali County hospital, Weinan City Fuping County hospital, Weinan City Fuping County hospital, Weinan City Heyang County hospital, Weinan City Huaxian people's hospital, Weinan City Huaxian hospital, Weinan Pucheng County traditional Chinese Medicine hospital, Weinan City first hospital, Baoji City Chencang hospital, Baoji City Jintai hospital, Baoji Fengxiang County traditional Chinese Medicine hospital, Baoji Fufeng County people's hospital, Baoji City Fufeng County traditional Chinese Medicine hospital, Baoji Mei County people's hospital, Baoji Mei County traditional Chinese Medicine hospital, Baoji City Qishan County traditional Chinese Medicine hospital, Baoji Feng County people's hospital, Baoji Central hospital, Baoji people's hospital, Baoji NO.987 hospital, Baoji third people's hospital, Baoji NO.409 hospital, Ankang City Langao County traditional Chinese Medicine hospital, Ankang City Pingli County hospital, Ankang City Shiquan County traditional Chinese Medicine hospital, Ankang City Ziyang County people's hospital, Hanzhong City Chenggu County hospital, Hanzhong City people's hospital, Hanzhong City Lueyang County people's hospital, Hanzhong City Ningqiang County Tianjin hospital, Ankang City people's hospital, Hanzhong City 3201 hospital, Yanan Huangling County people's hospital, Yan'an Zhidan County people's hospital, Yan'an Wuqi County people's hospital, Yan'an Luochuan County hospital, Yan'an Luochuan County people's hospital, Yan'an Fu County people's hospital, Yulin Hengshan County People's hospital, Yulin Jingbian County traditional Chinese Medicine hospital, Yulin city Mizhi County hospital, Yulin Suide County hospital, Yulin City Yuyang District People's hospital, Yulin City Zizhou County People's hospital, Yulin City Gaoxin hospital, Yulin Fugu County People's hospital, Yulin Qingjian County People's hospital, Fugu County traditional Chinese Medicine hospital, Xi'an Shaanxi provincial hospital, Xi'an Central hospital, Xi'an 986 hospital, Xi'an Tangdu hospital, Weinan center hospital, Hanzhong center hospital, Ankang traditional Chinese Medicine hospital.
