# Supplementary material for: Efficacy and safety of butylphthalide in secondary prevention of stroke: study protocol for a multicenter, real world trial based on Internet
Source: BMC Neurol. 2022 Aug 19;22:305. doi: 10.1186/s12883-022-02815-x (PMC9389750; doi:10.1186/s12883-022-02815-x)
Supplement: Supplementary file 2 — Additional file 2: Appendix 2.The informed consent form. [file 12883_2022_2815_MOESM2_ESM.docx]

**Appendix 2. Informed Consent Form**

**Informed Consent Form**

You has been confirmed that you have an ischemic stroke by your doctor. We will invite you to participate in a clinical trail called efficacy and safety of butylphthalide in secondary prevention of ischemic stroke: a multicenter, real world study based on Internet.This trial has been reviewed by the Ethics Committee of Northwestern University and agreed to conduct a clinical study.

Before you decide whether to participate in this study, please read the following as carefully as possible. It can help you understand this trial and why it is conducted, the procedure and duration of the study, and the benefits, risks and discomfort that may be brought to you in the trial. You can also discuss it with your relatives or friends, and ask your doctor for an explanation to help you make a decision.

1. **Research background and objective**

**1.1 Current status of disease burden and treatment**

Stroke is an acute cerebrovascular disease, which is a group of diseases caused by sudden rupture of cerebral blood vessels or blood flow into the brain caused by vascular blockage, including ischemic and hemorrhagic stroke. Stroke has become the first cause of death in China, and it is also the leading cause of disability among Chinese adults. Stroke has the characteristics of high morbidity, high mortality and high disability rate.

Stroke is a disease prone to recurrence. The latest Chinese Stroke Registration study data showed that the stroke recurrence of patients with ischemic stroke at 3 months, 6 months and 1 year after onset were as high as 10.9%, 13.4% and 14.7%. And stroke recurrence will aggravate the condition of patients, seriously affect the quality of life of stroke patients, and increase the stroke disability and mortality. At present, the most effective means to reduce stroke recurrence is scientific and reasonable stroke secondary prevention. Therefore, it is hoped that the study on the efficacy and safety of butylphthalide soft capsule in secondary prevention of stroke can provide clinical evidence for proving that the drug can effectively reduce stroke recurrence.

**1.2 Research objective**

The objective of this trial is to confirm the efficacy and safety of butylphthalide on secondary prevention of stroke patients through follow-up of the experimental group and the control group. It provides clinical evidence for NBP to prevent and treat stroke, and has certain significance for improving treatment and prognosis of patients.

**1.3 The number of participants included in the study**

8000 patients will be recruited from neurology departments of no less than 70 hospitals across the country. These hospitals have a high level of diagnosis and treatment and have the ability to carry out auxiliary examinations such as CT, MRI, and so on. Every doctor in each trial center is responsible for the enrollment and follow-up of the patients, and the follow-up period is expected to be one year.

**2. Who is suitable to participate in the study**

Subjects must meet the following criteria before they can be recruited:

1.Aged between 18 and 80 years;

2.Hospitalized patients with ischemic stroke diagnosed by clinical symptoms or signs. The clinical manifestations are acute onset of local or diffuse brain injury. The distribution of neurological impairment is consistent with that of injured blood vessels;

3.Imaging/laboratory confirmation of an acute vascular ischemic pathology;

4.Voluntarily participate and sign the informed consent form.

**3. Who is not suitable to participate in the research**

Patients who meet any of the following criteria will be excluded from the trial:

1. Discharged, transferred to another department or died within 24hours;
2. Severe heart, liver, kidney and other organs dysfunction or severe systemic diseases expected to survive less than one year;

3. Can not be followed up continuously;

4. Allergic to butylphthalide;

5. Pregnancy, lactation or planned pregnancy;

6. Severe mental disorders or dementia.

**4. What will you need to do if you participate in the study?**

4.1.Before you participate in the study, your doctor will ask you about your medical history, conduct imaging tests (including CT, MRI, etc.), laboratory tests (including blood routine, blood glucose, blood lipids, liver and kidney function, serum electrolytes, myocardial enzymes) and other tests to confirm the diagnosis of stroke.

If you are a qualified participant, you can voluntarily participate in the study and sign the informed consent form.

If you are unwilling to participate in the study, we will treat you as you wish.

4.2 If you volunteer to participate in the study, you will follow the following steps:

According to whether take butylphthalide or not，subjects will be divided into two groups：(1) butylphthalide group: 0.2g of butylphthalide capsules three times daily plus routine treatment (aspirin 50-300mg/d，Clopidogrel 75mg/d，etc).

1. control group: routine treatment (aspirin 50-300mg/d，Clopidogrel 75mg/d，etc).

The treatment duration is 90 consecutive days or more.

Follow-up will be arranged at months 1, 3, 6, 12 after discharge.The information will be collected during follow-up: patient's demographic information, stroke type, medical history, neurological impairment assessment (NIHSS score), activity of daily living assessment (modified Rankin score), imaging examination, laboratory examination and adverse events.

4.3. Other matters that need your cooperation

You should receive follow-up through the Internet on time. Your follow-up is very important because your doctor will judge whether your treatment is effective or not and adjust your medication accordingly.You should follow the doctor's instructions to take medicine, and fill in your medication record on time. You can use other drugs for related treatment during the study period. If you need other treatment, please contact your doctor in advance.

1. **The possible benefits of participating in the research**

Although there is evidence that butylphthalide has a definite effect on ischemic stroke, this is not guaranteed to be effective for you. Butylphthalide used in this study is not the only way to treat ischemic stroke. If butylphthalide is not effective for your condition, you can ask your doctor about possible alternative treatments.

1. **Possible adverse events, risks, discomfort and inconvenience to participate in the study**

This study is a prospective, multicenter, real-world clinical trial. Subjects will be assessed by uniformly trained neurologists to see if they meet the inclusion criteria. The stroke patients included in the study will be treated according to “*the secondary prevention of stroke in the Chinese guidelines for the diagnosis and treatment of Acute Cerebral Hemorrhage*” and “*the guidelines for the diagnosis and treatment of Acute Ischemic Stroke in China*”. We will choose specific treatment methods for different types of stroke according to your medical history, symptoms and signs.

If you have any discomfort during the study, you should inform your doctor in time, and he or she will make a judgment and adjust the appropriate treatment plan.

During the study, you need to be followed up on time, which may take up some time and cause inconvenience.

1. **Related expenses**

The doctor will try his best to prevent and treat the injury caused by this study. If an adverse event occurs in this trial, the committee of medical experts will judge whether it is related to basic therapeutic drugs. The organizer will provide treatment fees and corresponding economic compensation for the test-related damage in accordance with the provisions of China's *"Drug Clinical trial quality Management Code".*

The treatment and examination required for other diseases that you combine at the same time will not be free of charge.

1. **Personal information confidential**

Your medical records (research medical records / CRF, laboratory sheets, etc.) will be kept completely in the hospital. The doctor will record the test results on your medical record. Researchers, ethics committees and drug regulatory authorities will be allowed to access your medical records. Any public reports on the results of this study will not disclose your personal identity. We will make every effort to protect the privacy of your personal medical data within the limits permitted by law.

1. **How to get more information?**

You can ask any questions about this study at any time and get answers accordingly. If there is any new information during the study that may affect your willingness to continue the study, your doctor will inform you in time.

1. **You can choose to participate in the study and drop out of the study voluntarily.**
2. Whether to participate in the study or not depends entirely on your wishes. You can refuse to participate in the study or withdraw from the study at any time, which will not affect your relationship with your doctor, your routine treatment or other benefits. Depending on your condition, your doctor or researcher can suspend your participation in this study at any time during the study. If you want to drop out of the study, you may be asked about the reason and medication. You may also be required to undergo the necessary laboratory and physical examinations.
3. **What should you do now?**

It is up to you (and your family) to decide whether to participate in this study or not.

Please ask your doctor as much as possible before you decide to participate in the study.

Thank you for reading the above materials. If you decide to participate in this study, your doctor will arrange everything related to the study for you. Please keep this information.

Informed consent signature page

Name of clinical research project:__________________________________

Project undertaking unit:__________________________________________

Project collaboration unit:_________________________________________

Task No.:______________________________________________________

**Agree to the declaration**

I have read the above introduction to this study and have the opportunity to discuss and ask questions with my doctor about this study. All the questions I raised were answered satisfactorily.

I know the possible risks and benefits of participating in this study. I know that participation in the study is voluntary, and I confirm that I have had sufficient time to consider it, and I understand:

1.I can ask the doctor for more information at any time.

2.I can withdraw from this study at any time without discrimination or retaliation, and my medical treatment and rights and interests will not be affected.

3.I also know that if I drop out of the study, especially because of drugs, it will be beneficial to the whole study if I tell my doctor about the changes in my condition and complete the corresponding laboratory and physical examinations.

4.If I need to take other drugs due to changes in my condition, I will consult my doctor in advance.

5.I agree that the ethics committee of the drug regulatory department or the sponsor should consult my research materials.

6.I will get a signed and dated copy of the informed consent form.

7.Finally, I decided to agree to participate in this study and promised to follow the doctor's advice as much as possible.

**Patient signature:__________________ Date:____________**

**Phone numbers:__________________**

I confirm that I have explained to the patient the details of this trial, including its rights and possible benefits and risks, and give him a copy of the signed informed consent form.

**Doctor signature:__________________ Date:____________**

**Phone numbers:__________________**
